# EMBASE search strategies for identifying methodologically sound diagnostic studies for use by clinicians and researchers

**DOI:** 10.1186/1741-7015-3-7

**Published:** 2005-03-29

**Authors:** Nancy L Wilczynski, R Brian Haynes, the Hedges Team

**Affiliations:** 1 Health Information Research Unit, Department of Clinical Epidemiology and Biostatistics, McMaster University, Hamilton, Ontario, L8N 3Z5 Canada; 2 Health Information Research Unit, Department of Clinical Epidemiology and Biostatistics, Department of Medicine, McMaster University, Hamilton, Ontario, L8N 3Z5 Canada

## Abstract

**Background:**

Accurate diagnosis by clinicians is the cornerstone of decision making for recommending clinical interventions. The current best evidence from research concerning diagnostic tests changes unpredictably as science advances. Both clinicians and researchers need dependable access to published evidence concerning diagnostic accuracy. Bibliographic databases such as EMBASE provide the most widely available entrée to this literature. The objective of this study was to develop search strategies that optimize the retrieval of methodologically sound diagnostic studies from EMBASE for use by clinicians.

**Methods:**

An analytic survey was conducted, comparing hand searches of 55 journals with retrievals from EMBASE for 4,843 candidate search terms and 6,574 combinations. All articles were rated using purpose and quality indicators, and clinically relevant diagnostic accuracy articles were categorized as 'pass' or 'fail' according to explicit criteria for scientific merit. Candidate search strategies were run in EMBASE, the retrievals being compared with the hand search data. The proposed search strategies were treated as "diagnostic tests" for sound studies and the manual review of the literature was treated as the "gold standard." The sensitivity, specificity, precision and accuracy of the search strategies were calculated.

**Results:**

Of the 433 articles about diagnostic tests, 97 (22.4%) met basic criteria for scientific merit. Combinations of search terms reached peak sensitivities of 100% with specificity at 70.4%. Compared with best single terms, best multiple terms increased sensitivity for sound studies by 8.2% (absolute increase), but decreased specificity (absolute decrease 6%) when sensitivity was maximized. When terms were combined to maximize specificity, the single term "specificity.tw." (specificity of 98.2%) outperformed combinations of terms.

**Conclusion:**

Empirically derived search strategies combining indexing terms and textwords can achieve high sensitivity and specificity for retrieving sound diagnostic studies from EMBASE. These search filters will enhance the searching efforts of clinicians.

## Background

Accurate diagnosis is essential for both the clinical care of patients and research about disease conditions. Clinicians increasingly use online access to evidence in the course of clinical care as well as for continuing education and research [1]. For most clinicians and researchers the current best evidence published in health care journals is usually first widely accessible through major biomedical databases such as MEDLINE and EMBASE. However, information retrieval in these databases can be problematic due to the scatter of relevant articles across a broad array of journals, the very dilute concentration of high quality, relevant studies in a very large database, and the inherent limitations of indexing in any large bibliographic database, amplified by clinicians' lack of search skills [2]. EMBASE searches, for example, take place in the milieu of over 9 million citations from over 4,600 journals with between 6,000 and 8,000 citations added weekly [3].

Researchers have developed search strategies to assist clinicians with searching, the majority of which have been developed for MEDLINE when searching for therapy and review articles [4-10]. More recently, search strategies have been reported for the retrieval of diagnostic studies in MEDLINE [11-14]. This is an important development because clinicians must be able to efficiently retrieve the increasing amount of innovation and new knowledge concerning diagnosis and the burgeoning number of validated treatments for specific conditions that are contained in these large electronic databases. Using search strategies or filters can assist clinicians with this retrieval. In addition to searching MEDLINE, clinicians may wish to search other electronic databases such as EMBASE to cover their topic of interest more comprehensively. EMBASE is complementary to MEDLINE in that EMBASE provides greater coverage of the European and non-English language publications and provides broader coverage in such areas as psychiatry and toxicology [3].

In the early 1990s, our group at McMaster University developed search filters for use by clinicians and researchers on a small subset of 10 journals and for 4 types of journal articles (therapy, diagnosis, prognosis and causation [etiology]) [15,16]. This research was updated and expanded using data from 161 journals indexed in MEDLINE from the publishing year 2000 [17-20]. These search strategies have been adapted for use in the Clinical Queries interface of MEDLINE . Clinicians can easily access and use these search strategies by going to the Clinical Queries page in PubMed. We now report the extension of this research for EMBASE, including the information retrieval properties of single terms and combinations of terms for maximizing the sensitivity and specificity of identifying methodologically sound primary (original) studies on the diagnosis of health disorders. These search strategies will assist clinicians and researchers when searching for relevant, high-quality articles in EMBASE.

## Methods

We compared the retrieval performance of methodological search terms and phrases in EMBASE with a manual review of each article for each issue of 55 journal titles for the year 2000. Overall, research staff hand-searched 170 journal titles. These journals were chosen based on recommendations of clinicians and librarians, Science Citation Index Impact Factors provided by the Institute for Scientific Information, and the ongoing assessment of their yield of studies and reviews of scientific merit and clinical relevance for the disciplines of internal medicine, general medical practice, mental health, and general nursing practice (list of journals provided by the authors upon request). Of these 170 hand-searched journals, 135 were indexed in EMBASE. Search strategies were developed using a 55-journal subset chosen based on those journals that had the highest number of methodologically sound studies, that is, studies that clinicians should be using when making patient care decisions. This selection enriches the sample of target articles, improving the precision of estimates of search term performance and simplifying data processing, but is unlikely to bias the estimates of the sensitivity and specificity of search terms.

We compiled an initial list of search terms, including index terms and textwords from clinical studies. Input was then sought from clinicians and librarians in the United States and Canada through interviews of known searchers, and requests at meetings and conferences. We compiled a list of 5,385 terms of which 4,843 were unique and 3,524 returned results (list of terms tested provided by the authors upon request). Examples of the search terms tested are 'criterion standard', 'cut point', 'sensitivity', and 'ROC curve', all as textwords; 'diagnosis', the index term, and the index term 'diagnostic test', exploded (that is, including all of this term's indexing subheadings).

As part of a larger study [21], research staff performance was rigorously calibrated before reviewing the journals and inter-rater agreement for identifying the purpose of articles was 81% beyond chance (kappa statistic, 95% confidence interval (CI) 0.79 to 0.84). Inter-rater agreement for which articles met all methodological criteria was 89% (CI 78% to 99%) beyond chance [21]. Six research assistants then hand-searched all articles in each issue of the 55 journals and applied methodological criteria to determine whether the article was methodologically sound for evaluation of a diagnostic test. The methodological criteria applied for studies of diagnosis were as follows: Inclusion of a spectrum of participants; objective diagnostic ("gold") standard or current clinical standard for diagnosis; participants received both the new test and some form of the diagnostic standard; interpretation of diagnostic standard without knowledge of test result and vice versa; and analysis consistent with study design.

The proposed search strategies were treated as "diagnostic tests" for sound studies and the manual review of the literature was treated as the "gold standard". We determined the sensitivity, specificity, precision and accuracy of each single term and combinations of terms in EMBASE using an automated process. Borrowing from the concepts of diagnostic test evaluation and library science, sensitivity for a given topic is defined as the proportion of high quality articles for that topic that are retrieved; specificity is the proportion of low quality articles not retrieved; precision is the proportion of retrieved articles that are of high quality; and accuracy is the proportion of all articles that are correctly classified [22].

Individual search terms with sensitivity > 25% and specificity > 75% for a given purpose category were incorporated into the development of search strategies that included 2 or more terms. All combinations of terms used the Boolean OR, for example, "predict.tw. OR specificity.tw.". The Boolean AND was not used because this strategy invariably compromised sensitivity. For the development of multiple-term search strategies to optimize either sensitivity or specificity, we tested all 2-term search strategies with sensitivity at least 75% and specificity at least 50%. For optimizing accuracy, 2-term search strategies with accuracy > 75% were considered for multiple-term development. In the development of diagnosis search filters, 6,574 search strategies were tested.

In addition to developing search strategies using the Boolean approach described above, we also evaluated the potential for improving performance using logistic regression. Two approaches were taken. First, we took the top performing Boolean search strategies and ORed additional terms to these base strategies using stepwise logistic regression. The level of significance for entering and removing search terms from the model was 0.05. Adding terms to the model stopped when the increase in the area under the ROC curve was < 1%. Second, we developed search strategies from scratch with stepwise logistic regression using these same cut-off values. Both logistic regression approaches were compared with the Boolean approach to search strategy development when developing strategies for treatment articles and prognostic articles for MEDLINE. Treatment and prognosis were chosen because they represented the best and the worst cases for MEDLINE search strategy performance. For both purpose categories, the logistic regression approaches to developing search strategies did not improve performance compared with search strategies developed using the Boolean approach described above. Thus, for subsequent purpose categories, including diagnosis and databases, including EMBASE, the Boolean approach was used for search strategy development.

We also tested search strategies published by other researchers for detecting diagnosis studies.

## Results

Indexing information was downloaded from EMBASE for 27,769 articles from the 55 hand-searched journals. Of these, 433 were classified as diagnosis, of which 97 (22.4%) were methodologically sound. Search strategies were developed using all 27,769 articles. Thus, the strategies were tested for their ability to retrieve articles about high quality diagnosis studies from all other articles, including both low quality diagnosis studies and all non-diagnosis studies.

Table 1 shows the best single term for high-sensitivity, high-specificity, and best balance of sensitivity and specificity. The single term, "di.fs." (Ovid syntax for diagnosis as a floating subheading) produced the best sensitivity of 91.8% while keeping specificity at 76.4%. Specificity was maximized at 98.2% using the single term "specificity.tw.", but this was achieved at the expense of sensitivity, 62.9%. The single term "diagnos:.mp." (Ovid syntax for the appearance of "diagnos:" in any one of the title, abstract or subject headings), produced the optimal balance between sensitivity (89.7%) and specificity (84.7%).

**Table 1:** Single Term with the Best Sensitivity, Best Specificity, and Best Optimization of Sensitivity and Specificity for Detecting Studies of Diagnosis in EMBASE in 2000. Values are percentages (95% confidence intervals).

Search term OVID search*	**Sensitivity (n = 97)**	**Specificity (n = 27672)**	**Precision†**	**Accuracy (n = 27769)**
**Best sensitivity **(keeping specificity ≥ 50%) di.fs.	91.8 (86.3 to 97.2)	76.4 (75.9 to 76.9)	1.4 (1.1 to 1.6)	76.5 (76.0 to 77.0)
**Best specificity **(keeping sensitivity ≥ 50%) specificity.tw.	62.9 (53.5 to 72.5)	98.2 (98.1 to 98.4)	11.0 (8.4 to 13.6)	98.1 (97.9 to 98.3)
**Best Optimization of Sensitivity & Specificity‡** diagnos:.mp.	89.7 (83.6 to 95.7)	84.7 (84.3 to 85.2)	2.0 (1.6 to 2.4)	84.8 (84.3 to 85.2)

*Search strategies are reported using Ovid's search engine syntax for EMBASE. †Denominator varies by row. ‡Based on the lowest possible absolute difference between sensitivity and specificity. di = diagnosis; fs = floating subheading; tw = textword (word or phrase appears in title or abstract); : = truncation; mp = multiple posting – term appears in title, abstract, or subject heading. Sensitivity = the proportion of high quality articles for that topic that are retrieved; specificity = the proportion of low quality articles not retrieved; precision = the proportion of retrieved articles that are of high quality; accuracy = the proportion of all articles that are correctly classified.

Combinations of terms with the best results for sensitivity, specificity and optimization of sensitivity and specificity are shown in Table 2. Combinations of terms improved on single search term performance for sensitivity. The 3-term search strategy, "di.fs. OR predict:.tw. OR specificity.tw.", achieved a sensitivity of 100% with a specificity at 70.4%. The single term "specificity.tw." had the highest specificity, outperforming all 2- and 3-term combinations. A 3-term combination resulted in the optimization strategy achieving slightly above 89% for both sensitivity and specificity (Table 2).

**Table 2:** Combination of Terms with the Best Sensitivity, Best Specificity, and Best Optimization of Sensitivity and Specificity for Detecting Studies of Diagnosis in EMBASE in 2000. Values are percentages (95% confidence intervals).

Search Strategyn OVID search*	**Sensitivity (n = 97)**	**Specificity (n = 27672)**	**Precision†**	**Accuracy (n = 27769)**
**Best Sensitivity **(keeping specificity ≥ 50%) di.fs. OR predict:.tw. OR specificity.tw.	100.0 (100.0 to 100.0)	70.4 (69.8 to 70.9)	1.2 (0.9 to 1.4)	70.5 (69.9 to 71.0)
**Small drop in sensitivity with a substantive gain in specificity** diagnos:.mp. OR predict:.tw. OR specificity.tw.	96.9 (93.5 to 100.0)	78.2 (77.7 to 78.7)	1.5 (1.2 to 1.8)	78.3 (77.8 to 78.8)
**Best Specificity **(keeping sensitivity ≥ 50%) specificity.tw.	62.9 (53.5 to 72.5)	98.2 (98.1 to 98.4)	11.0 (8.4 to 13.6)	98.1 (97.9 to 98.3)
**Small drop in specificity with a substantive gain in sensitivity** specificity.tw. OR accurac:.tw.	73.2 (64.4 to 82.0)	97.4 (97.2 to 97.5)	8.8 (6.9 to 10.8)	97.3 (97.1 to 97.5)
**Best Optimization of Sensitivity & Specificity‡** sensitiv:.tw. OR diagnostic accuracy.sh. OR diagnostic.tw.	89.7 (83.6 to 95.7)	91.6 (91.3 to 91.9)	3.3 (2.9 to 4.4)	91.6 (91.3 to 91.9)

*Search strategies are reported using Ovid's search engine syntax for EMBASE. †Denominator varies by row. ‡Based on the lowest possible absolute difference between sensitivity and specificity. di = diagnosis; fs = floating subheading; : = truncation; tw = textword (word or phrase appears in title or abstract); mp = multiple posting – term appears in title, abstract, or subject heading; sh = subject heading. Sensitivity = the proportion of high quality articles for that topic that are retrieved; specificity = the proportion of low quality articles not retrieved; precision = the proportion of retrieved articles that are of high quality; accuracy = the proportion of all articles that are correctly classified.

Slight modifications to the above-noted most sensitive and most specific search strategies led to some attractive trade-offs in sensitivity and specificity (Table 2). For instance, by replacing "di.fs" with "diagnos:.mp." in the most sensitive search strategy ("diagnos:.mp. OR predict:.tw. OR specificity.tw.") specificity increased (70.4% to 78.2%) at the price of a small decrease in sensitivity (100% to 96.9%). Additionally, by ORing "accurac:.tw." to "specificity.tw.", to the most specific search strategy, sensitivity increased by 10.2% (62.9% to 73.2%) with a small decrease in specificity (98.2% to 97.4%).

Our search strategies were simpler and compared well with two previously published strategies by Bachmann and colleagues for retrieving diagnostic test studies from EMBASE [23]. The most sensitive search reported by Bachmann and colleagues, an 8-term strategy, had a sensitivity of 96.9% in our database compared with 100% for our 3-term strategy (difference 3.1%, 95% CI -0.8% to 8.7%) (Table 3). The most specific search reported by Bachmann and colleagues, a 2-term strategy, had a specificity of 90.9% in our database, compared with 98.2% for our 1-term strategy, but our strategy was less sensitive (62.9 vs. 79.4, difference 16.5%, CI 3.8% to 28.9%). Unlike Bachmann's study, our study evaluated the methodological rigor of diagnosis studies, and thus the performance of search strategies compared here is for detecting methodologically sound diagnostic studies.

**Table 3:** Comparison of previously published search strategies with search strategies developed our database. Values are percentages.

Search Strategy OVID search*	**Sensitivity**	**Specificity**	**Precision**	**Accuracy**
**Bachmann's most sensitive search [22]** sensitiv:.tw. OR detect:.tw. OR accura:.tw. OR specific:.tw. OR reliab:.tw. OR positive.tw. OR negative.tw. OR diagnos:.tw.	100.0	Not reported		Not reported
**Bachmann's strategy tested in our database**	96.9	72.3	1.2	72.4
**Our most sensitive search** di.fs. OR predict:.tw. OR specificity.tw.	100.0	70.4	1.2	70.5
**Difference (our strategy – Bachmann) 95% CI for the difference**	3.1 -0.8 to 8.7†	-1.9 -2.7 to -1.2‡	0	-1.9 -2.7 to -1.2‡
**Bachmann's most specific search** sensitiv:.tw. OR detect:.tw.	73.7	Not reported		Not reported
**Bachmann's strategy tested in our database**	79.4	90.9	3.0	90.8
**Our most specific search** specificity.tw.	62.9	98.2	11.0	98.1
**Difference (our strategy – Bachmann) 95% CI for the difference**	-16.5 -28.9 to -3.8‡	7.3 7.0 to 7.7‡	8.0 5.6 to 11.0‡	7.3 6.9 to 7.7‡

*Search strategies are reported using Ovid's search engine syntax for EMBASE. †Differences are not statistically significant. ‡Differences are statistically significant. : = truncation; tw = textword (word or phrase appears in title or abstract); di = diagnosis; fs = floating subheading. Sensitivity = the proportion of high quality articles for that topic that are retrieved; specificity = the proportion of low quality articles not retrieved; precision = the proportion of retrieved articles that are of high quality; accuracy = the proportion of all articles that are correctly classified.

## Discussion

Our study documents search strategies for use by clinicians and researchers that can help discriminate relevant, high-quality studies from lower quality studies of the diagnosis of health disorders and articles that are not about diagnosis. Those interested in all sound articles on diagnosis, for example researchers conducting systematic reviews of diagnostic tests, will be best served by the most sensitive search. If systematic reviewers wish to include diagnostic test articles that fail the methodological criteria we set, they will still be well served by starting with this strategy: in addition to retrieving all sound studies, the suboptimal specificity (70.4%) of our most sensitive search strategy means the many lower quality diagnostic test studies will also be retrieved. Reviewers may then use additional means to ensure that all pertinent studies are retrieved. Those with little time on their hands who are looking for a few good articles on diagnosis, most likely clinicians, will probably be best served by the most specific strategies. Clinicians could further broaden their search by using the strategies that optimize sensitivity and specificity while minimizing the difference between the two as these strategies provide the best separation of "hits" (target citations) from "false drops" (undesired citations) but do so without regard for whether sensitivity and specificity are affected.

In all cases precision was low. This is the inevitable result of a low proportion of relevant studies for a given purpose in a very large, multipurpose database. This means that clinicians and researchers will continue to need to invest their time in discarding irrelevant retrievals. While low precision in searching can be of concern, the low values here should not be over-interpreted: we did not limit the searches by clinical content terms, as would usually be the case in clinical searches. Precision might be enhanced by combining search strategies in these tables with additional methodological terms using the Boolean 'AND NOT', thereby reducing the possibility of retrieving studies of lower methodological quality; however, this may decrease the sensitivity of the searches. Precision might also be increased by combining search strategies with content specific terms (e.g., "diabetes") or journal subsets using the Boolean 'AND' thus reducing the volume of literature searched. The next phases of our project will focus on finding better search strategies through using more sophisticated strategies as outlined above.

Comparing the diagnostic search strategies developed for EMBASE with those that we developed for MEDLINE [19], we found that the single term "specificity.tw." was the top performer for specificity in both databases and that this term outperformed 2- and 3-term strategies. Additionally, we found that textwords outperformed most index terms for sensitivity and specificity. The only index term that was a top performer was "di.fs." or "di.xs.", which was the case for both databases. Although there are many differences between EMBASE and MEDLINE, some basic similarities are apparent, as just described.

Comparing our diagnostic search strategies developed for EMBASE with those previously published [23], our strategies had fewer terms and performed at least as well.

## Conclusion

Selected combinations of indexing terms and textwords can achieve high sensitivity or specificity in retrieving diagnosis studies cited in EMBASE. The reported search strategies will assist both clinicians and researchers when attempting the retrieve relevant, high-quality diagnostic articles.

## Competing interests

The author(s) declare that they have no competing interests.

## Authors' contributions

NLW and RBH prepared grant submissions in relation to this project. Both authors drafted, commented on and approved the final manuscript. Both authors also supplied intellectual content to the collection and analysis of the data. NLW participated in the data collection and both authors were involved in data analysis and staff supervision.

## Pre-publication history

The pre-publication history for this paper can be accessed here:


